# Receptor oligomerization: from early evidence to current understanding in class B GPCRs

**DOI:** 10.3389/fendo.2012.00175

**Published:** 2013-01-04

**Authors:** Stephanie Y. L. Ng, Leo T. O. Lee, Billy K. C. Chow

**Affiliations:** Endocrinology, School of Biological Sciences, The University of Hong KongHong Kong, China

**Keywords:** GPCR, class B, secretin receptor, oligomerization, BRET

## Abstract

Dimerization or oligomerization of G protein-coupled receptors (GPCRs) are known to modulate receptor functions in terms of ontogeny, ligand-oriented regulation, pharmacological diversity, signal transduction, and internalization. Class B GPCRs are receptors to a family of hormones including secretin, growth hormone-releasing hormone, vasoactive intestinal polypeptide and parathyroid hormone, among others. The functional implications of receptor dimerization have extensively been studied in class A GPCRs, while less is known regarding its function in class B GPCRs. This article reviews receptor oligomerization in terms of the early evidence and current understanding particularly of class B GPCRs.

## INTRODUCTION

G protein-coupled receptors (GPCRs) comprise the largest subset of cell surface receptors which transduce signals via coupling to heterotrimeric G proteins and are extensively involved in the fine-tuning of physiological processes. They constitute at least 3% of the human genome and have great pathophysiological importance with an abundance of information indicating their dysfunction to be associated with diseases such as diabetes, visual disability, and chronic inflammation. As a result, at least 30–40% of pharmaceutical drugs developed are targeted at GPCRs. Traditionally, GPCRs were recognized to elicit physiological responses via coupling as monomeric units to G proteins in a 1:1 stoichiometric ratio. However there is now a growing body of evidence confirming that GPCRs can self-associate or associate with different receptors to form homo-and/or hetero-oligomers. With the wealth of information recently discovered, oligomerization has been implicated to play important roles in maturation, cell surface delivery, signaling, and internalization of GPCRs. The concept of oligomerization is groundbreaking as it not only widens our perspective in understanding the molecular determinants to receptor regulation and function, but also provides new opportunities in the development of personalized drug treatments. In recent years, the small 15-member class B [secretin (SCT) or class II] GPCRs are emerging as oligomerization candidates with efforts contributed predominantly by Laurence Miller’s and Dominik Schelshorn’s groups. In this article, we will provide an overview of the history of oligomerization and review the available information on class B GPCRs and its functional implications.

## EARLY EVIDENCE OF GPCR DIMERIZATION BY PHARMACOLOGICAL AND BIOCHEMICAL APPROACHES

Although GPCR oligomerization is now a widely accepted phenomenon with modern non-radiative techniques such as resonance energy transfer (RET) strategies that are effective in demonstrating homo- and/or hetero-oligomer interactions, early clues to their existence were often indirect and overlooked. In 1975, a binding experiment of a potent antagonist to β_2_-adrenergic receptors (ADRB2) in frog erythrocyte membranes led to findings of negative cooperativity among binding sites (Limbird et al., 1975; Limbird and Lefkowitz, 1976). This was pioneering evidence for oligomerization and can be better explained as site–site interactions amongst ADRB2 oligomers based on today’s knowledge. Soon after, radiation inactivation, cross-linking and co-immunoprecipitation studies have also provided biochemical evidence complementing earlier observations of oligomerization. For example, immunoaffinity chromatography and sodium dodecyl sulfate-polyacrylamide gel electrophoresis (SDS-PAGE) showed the subunit molecular weight of mammalian lung ADRB2 to be 59 kDa, while radiation inactivation of ADRB2 resulted in a functional subunit molecular weight of 109 kDa (Fraser and Venter, 1982). Taken together, the contrasting molecular sizes indicate that ADRB2s exist in the membrane as dimers of two subunits (Fraser and Venter, 1982). Similar suggestions have also been made for the rat liver membrane α_1_-adrenergic receptor (ADRA1; Venter et al., 1984) and human platelet α_2_-adrenergic receptor (ADRA2; Venter et al., 1983) which each have molecular masses of 160 kDa. Alternatively, cross-linking experiments, using photoaffinity labeling reagents, cell permeable cross-linkers, agonists, and antagonists (Capponi and Catt, 1980; Paglin and Jamieson, 1982; Guillemette and Escher, 1983; Rogers, 1984; Carson et al., 1987; Rondeau et al., 1990; Siemens et al., 1991) have been used to study the dimeric nature of angiotensin II (ANGII) receptors. Using similar methods, other GPCRs such as the dopamine D_2_ (Ng et al., 1996), calcium-sensing (Bai et al., 1998), and chemokine (Rodriguez-Frade et al., 1999) receptors have also been demonstrated to exist as dimeric units. Other experiments such as photoaffinity labeling of MRs from various brain regions and the heart have also provided evidence for the presence of inter convertible dimers and tetramers (Avissar et al., 1983). The idea of higher order oligomerization was further supported by comparable findings of thyrotropin hormone receptor complexes (Gennick et al., 1987).

In the 1990s, another line of evidence demonstrating GPCR oligomerization stemmed from *trans*-complementation, and dominant-negative and -positive effect studies on co-expression of chimeric and mutant receptor constructs. One of the first experiments of these series was performed in 1993 by co-expression of chimeric muscarinic and adrenergic receptors composed of their C-terminal units (Maggio et al., 1993). Although no binding activity was detected when the chimeras were expressed individually, co-expression restored ligand binding to a level comparable to wild-type receptors and allowed agonist-dependent phospholipase C (PLC) activation (Maggio et al., 1993). These observations were explained as molecular interaction of the chimeras restoring functionality in a heteromeric complex (Maggio et al., 1993). Other *trans*-complementation studies for GPCRs such as the ANGII type 1a (AGTR1a; Monnot et al., 1996), somatostatin (SST – SST_1_ and SST_5_; Rocheville et al., 2000) and dopamine (D_2_ and D_3_; Scarselli et al., 2001) receptors have also provided useful information in support of GPCR oligomerization. This concept was further explored in experiments showing dominant-negative or -positive effects such those observed with the antidiuretic V2 vasopressin (Zhu and Wess, 1998) and ADRB2 (Hebert et al., 1998), respectively. Although there are reports of different functional effects, the various studies described are useful in offering more clues to molecular interactions amongst GPCRs.

Though earlier experiments provided a large pool of information suggesting GPCR oligomerization, there was still no direct evidence to support this concept. Early western blot studies often showed presence of immuno-reactive bands with molecular masses two or more times greater than a single receptor (Harrison and van der Graaf, 2006). Western blot was later performed in combination with immunoprecipitation using antibodies to endogenous or epitope tagged-receptors, providing for the first time direct evidence of GPCR interaction. Using this technique, homodimerization of ADRB2 (Hebert et al., 1996) was demonstrated. For example, cross-species oligomerization was also detected in select serotonin receptors with lysophosphatidic acid receptors 1 and 3 and γ-aminobutyric acid B_2_ receptors (Salim et al., 2002), dopamine D_1_ and D_2_ receptors (Lee et al., 2004), and α_1b_ and β_2_ adrenergic receptors (Uberti et al., 2005).

## RET APPROACH IN GPCR OLIGOMERIZATION

Since the days of radioactive inactivation experiments, the concept of GPCR oligomerization has been confirmed with emergence of biophysical data providing more in-depth evidence. In the last decade, RET techniques have gained popularity and are one of the best resolution strategies developed for direct study of GPCR interactions. The basis of these techniques lies in non-radiative energy transfer from an excited “donor” molecule to an “acceptor” (Wu and Brand, 1994). Bioluminescence RET (BRET) and fluorescence RET (FRET) are two of the most widely used RET approaches. BRET relies on a naturally occurring biophysical process in marine species between a luminescent donor and fluorescent acceptor, while only fluorescent molecules are utilized in FRET (Wu and Brand, 1994). These have provided advantages over traditional biochemical methods including high signal to noise ratios, use of intact cells, precise targeting of fusion receptors and actual quantification of the proportion and type of GPCR oligomers formed, and therefore are currently the most commonly used methods. Based on these approaches, a wide range of GPCRs have since been identified to function as oligomers, including the yeast α-factor (Overton and Blumer, 2000), dopamine D_2_ (Wurch et al., 2001), thyrotropin (Latif et al., 2001, 2002), opioid (Ramsay et al., 2002), neuropeptide Y (Dinger et al., 2003), melatonin (Ayoub et al., 2004), adrenergic (Mercier et al., 2002; Ramsay et al., 2002; Carrillo et al., 2004), and chemokine (Percherancier et al., 2005) receptors. Despite the successes in identifying various oligomers, there are several drawbacks of the BRET and FRET techniques. With regards to these aforementioned RET techniques, a primary concern is that the signals generated cannot be used to discriminate between non-mature and mature proteins within the various cellular locations (e.g., intracellular complexes and plasma membrane; Gandia et al., 2008; Cottet et al., 2011). Therefore, the information provided is essentially limited and more steps may be needed to address this concern. Moreover, another drawback of BRET and FRET is potential bleed-through artifacts which may be due to spectral overlaps, substrate instability, and auto-luminescence from serum containing medium, which together skew the interpretation of the results (Pfleger et al., 2006; Gandia et al., 2008). Therefore, the careful use of appropriate controls and fluorescent molecules need to be considered when using these RET techniques for the study of oligomerization. More recently, homogeneous time-resolved fluorescence (HTRF) which combines FRET with time-resolved measurements has also been considered. HTRF provides the advantage of increasing assay sensitivity and accuracy via replacing donor molecules with rare-earth lanthanides (e.g., europium cryptate) whose properties include non-auto-fluorescence and long emission half-lives (Degorce et al., 2009). Using HTRF, a number of receptors have been identified to dimerize, e.g., GABA (Maurel et al., 2004) and metabotropic glutamate-like receptors (Rondard et al., 2006; Brock et al., 2007) and oligomerize, e.g., dopamine D_2_ (Guo et al., 2008) and histamine H4 (Albizu et al., 2006) receptors. Fluorescence life-time imaging microscopy (FLIM) is also useful in overcoming the drawback of the use of intact cells and bleed-through in BRET and FRET assays. Moreover, FLIM can also provide useful information on the proportion of non-dimerizing and dimerizing partners and also visual images indicating where the signals are localized, for example the dimerization of the transcription factor CCAAT and enhancer binding protein-α in live mouse pituitary cell nuclei (Sun et al., 2011).

## OLIGOMERIZATION OF CLASS B GPCRs

Although techniques in studying oligomerization have existed for more than three decades, information with regards to the class B GPCRs is still in its infancy and largely contributed by the newer RET techniques. In class A GPCRs, several important interfaces including the transmembrane (TM) domains have been identified to contribute toward oligomerization. Moreover, oligomerization has also been shown to play a role in modulating function, via altering parameters such as ligand binding, signaling, and trafficking. Though the SCT receptor (SCTR) remains to be the most extensively studied class B GPCR, information regarding other class B GPCRs continues to grow. Collectively, the SCT superfamily of hormones and receptors represent important drug targets due to their physiological roles in glucose homeostasis, feeding behavior, and vascular contractility. They are characterized by a large N-terminal extracellular domain (ECD) containing six cysteine residues for disulfide bond formation and *N*-glycosylation sites for receptor conformation and ligand binding. The N-terminal ectodomain is linked to seven hydrophobic TM domains arranged in α-helical bundles connected by three exoloops, three endoloops, and a short cytosolic C-terminus (Miller et al., 2007). Currently, there are intense efforts to link the various structural domains to oligomerization in hopes of unraveling the specific molecular determinants that may affect certain pathological conditions. Taken together, information regarding the oligomerization of class B GPCRs will be highly valuable in enhancing the specificity of future drug design.

### SECRETIN RECEPTORS

The SCTR was first cloned from the rat in 1991 (Ishihara et al., 1991) and is now recognized as one of the most extensively studied class B GPCRs, and the first that was demonstrated to have the ability to form oligomers. By investigating wild-type and exon-3 splice variants using confocal imaging and BRET, SCTR has been shown to be capable of homo- and heterodimerization (Ding et al., 2002a,b; **Tables 1 and 2**). When expressed alone, the exon-3 splice variant receptors lack SCT binding and signaling activity but are able to traffic normally to the cell surface. However, co-expression of the variant with the wild-type SCT results in a dominant-negative effect, indicating direct physical interaction. Since the variant receptor is predominantly expressed, comprising up to 70% of SCTRs in gastrinoma, over the wild-type receptor, heterodimerization has been suggested to be an underlying factor in facilitating tumor growth through its dominant-negative effects. This study in 2002 was a breakthrough for class B GPCRs, showing the first physiological relevance of class B GPCR oligomerization. Taken together with data from subsequent studies, SCTR homodimerization is now recognized to occur, independent of ligand binding (Ding et al., 2002a; Harikumar et al., 2006).

**Table 1:** Homodimerization of class B GPCRs.

Homodimer	Technique	Key domain	Reference
SCTR/SCTR	Morphological FRET BRET	GxxxG motif independent Lipid-exposed TM4 residues (human: Gly^243^, Ile^247^)	Ding et al. (2002a), Harikumar et al. (2006), 2007, (2008a), Lisenbee and Miller (2006)
GCGR/GCGR	BRET	n/a	Roed et al. (2012), Schelshorn et al. (2012)
GLP1R/GLP1R	BRET	n/a	Schelshorn et al. (2012), Whitaker et al. (2012)
GLP2R/GLP2R	BRET	n/a	Schelshorn et al. (2012)
GIPR/GIPR	BRET	n/a	Schelshorn et al. (2012), Whitaker et al. (2012)
VIPR1/ VIPR1	Immunohistochemistry Co-immunoprecipitation Morphological FRET BRET	n/a	Harikumar et al. (2006)
VIPR2/VIPR2	Morphological FRET BRET	n/a	Harikumar et al. (2006)
ADCYAP1R1/ADCYAP1R1	Time-resolved FRET	n/a	Maurel et al. (2008)
GHRHR/GHRHR	Co-immunoprecipitation		McElvaine and Mayo (2006)
PTHR1/PTHR1	Crystallography FRET BRET	ECD α-helix (human: Ile^135^, Arg^179^, Val^183^, Leu^187^)	Pioszak et al. (2010)
CALCR/CALCR	Co-immunoprecipitation FRET BRET	Lipid-exposed TM4 residues (human: Arg^236^, Val^250^, Thr^253^) C-terminal tail	Seck et al. (2003), Harikumar et al. (2010)
CALCRL/CALCRL	Co-immunoprecipitation FRET BRET	n/a	Heroux et al. (2007)
CRHR1/CRHR1	FRET BRET	Pseudo signal peptide (based on rat CRHR2a: Asn^13^ prevents oligomerization)	Kraetke et al. (2005), Young et al. (2007), Teichmann et al. (2012)

*Techniques used for study and domains indicated to be important are described. SCTR, secretin receptor; GCGR, glucagon receptor; GLP1R, glucagon-like peptide 1 receptor; GLP2R, glucagon-like peptide 2 receptor; GIPR, glucose-dependent insulinotropic polypeptide receptor; VIPR1, vasoactive intestinal peptide (VIP) receptor 1; VIPR2, VIP receptor 2; ADCYAP1R1, PACAP type 1 receptor; GHRHR, growth hormone-releasing hormone receptor; PTHR1, parathyroid hormone receptor type 1; CALCR, calcitonin receptor; CALCRL, CALCR-like receptor; CRHR, corticotropin-releasing hormone receptor; FRET, fluorescence resonance energy transfer; BRET, bioluminescence resonance energy transfer; n/a, information not available.*

**Table 2:** Heterodimerization of class B GPCRs.

Heterodimer	Technique	Key domain	Reference
SCTR/GLP1R	BRET	n/a	Harikumar et al. (2008b)
SCTR/GLP2R	BRET	n/a	Harikumar et al. (2008b)
SCTR/VIPR1	Morphological FRET BRET	n/a	Harikumar et al. (2006), 2008b
SCTR/VIPR2	Morphological FRET BRET	n/a	Harikumar et al. (2006), 2008b
SCTR/GHRHR	BRET	n/a	Harikumar et al. (2008b)
SCTR/PTHR1	BRET	n/a	Harikumar et al. (2008b)
SCTR/PTHR2	BRET	n/a	Harikumar et al. (2008b)
SCTR/CALCRL	BRET	n/a	Harikumar et al. (2008b)
GCGR/GLP1R	BRET	n/a	Roed et al. (2012), Schelshorn et al. (2012)
GCGR/GLP2R	BRET	n/a	Schelshorn et al. (2012)
GCGR/GIPR	BRET	n/a	Schelshorn et al. (2012)
GLP1R/GIPR	BRET	n/a	Schelshorn et al. (2012), Whitaker et al. (2012)
GLP2R/GIPR	BRET	n/a	Schelshorn et al. (2012)
VIPR1/VIPR2	Co-immunoprecipitation BRET	n/a	Harikumar et al. (2006)

To decipher the molecular determinants of oligomerization, receptors with mutated N- and C- terminal domains were studied (Lisenbee and Miller, 2006). Mutant receptors, as expected, were unable to bind SCT or sort efficiently to the plasma membrane, but the ability to still produce BRET signals above background suggested that the TM spanning core alone was sufficient for oligomerization (Lisenbee and Miller, 2006). Moreover, SCTR dimerization was also found to be independent of the GxxxG helix–helix motif (Lisenbee and Miller, 2006), which is essential for oligomerization of class A receptors, such as the yeast α-factor (Overton et al., 2003) and β_2_ adrenergic (Salahpour et al., 2004) receptors. In addition, FRET signals within organelles of the receptor biosynthetic pathway including the endoplasmic reticulum, Golgi clusters, and plasma membrane were also detected, suggesting the occurrence of oligomerization even during the maturation of nascent molecules (Lisenbee and Miller, 2006). To further explore the domains responsible for homodimerization, TM peptide competition experiments have been performed. Of the seven TM peptides applied, TM4 was the only segment that disrupted BRET signals, suggesting it to be a functionally important interface for oligomerization (Harikumar et al., 2007). Using alanine mutagenesis, the contribution of TM4 in SCTR oligomers was pinpointed to two lipid-facing residues: Gly^243^ and Ile^247^ (Harikumar et al., 2007). Although no direct pathophysiological connection has been made regarding these new data, reduced signaling of monomeric forms suggests that SCTR dimers are necessary in optimizing functionality (Harikumar et al., 2007). Most recently, bimolecular luminescence complementation and BRET experiments have clarified SCTR’s existence as homodimers and not higher order oligomers (Harikumar et al., 2008a). Therefore, previous references to oligomerization of SCTRs should be referred to as homodimerization unless otherwise stated. Aside from homodimers, SCTRs have also been implicated to form heterodimers with a wide range of class B GPCRs, including the glucagon-like peptide 1 receptor (GLP1R), glucagon-like peptide 2 receptor (GLP2R), vasoactive intestinal peptide receptor 1 (VIPR1), vasoactive intestinal peptide receptor 2 (VIPR2), growth hormone-releasing hormone receptor (GHRHR), parathyroid hormone 1 receptor (PTHR1), parathyroid hormone 2 receptor (PTHR2), and calcitonin receptor-like (CALCRL; Harikumar et al., 2006, 2008b; **Table 2**).

### GCG, GLP1, GLP2, AND GIP RECEPTORS

Receptors for glucagon (GCG), glucagon-like peptide 1 (GLP1), glucagon-like peptide 2 (GLP2), and glucose-independent insulinotropic polypeptide (GIP) are well-known regulators of carbohydrate, fat, and protein metabolism. Oligomerization within the GCG subfamily of receptors was recently identified but with some controversy (Roed et al., 2012; Schelshorn et al., 2012). Homodimerization of each GCG family receptor member has been reported through BRET experiments (Schelshorn et al., 2012), however, these events remained unconfirmed with the lack of appropriate negative controls and the fact that another group was unable to repeat some of these results (Roed et al., 2012). Amongst the receptors, the GCG receptor (GCGR) and GIP receptor (GIPR) exhibited the strongest ability to homodimerize but BRET signals were slightly reduced upon application of GCG or GIP, while the GLP receptors were weakly responsive and unaffected by agonist stimulation (Schelshorn et al., 2012). Further studies with BRET saturation experiments confirmed GLP1R and GIPR homodimerization, with some agonist-induced reduction (Schelshorn et al., 2012). On the basis of these results, it was suggested that ligand binding leads to a decrease in affinity and conformational change of the homodimers (Schelshorn et al., 2012). In a similar study, the same BRET saturation results were obtained for the GLP1R, however no changes were observed when GLP1 was added (Roed et al., 2012). This was similarly the case for GCGRs (Roed et al., 2012). Taken together, it is highly likely that the GCG subfamily receptors are able to form homodimers (**Table 1**), while further studies with appropriate use of controls are necessary to solidify this conclusion.

Despite the controversy, heterodimerization of GLP1R and GIPR has been confirmed by BRET saturation and kinetic experiments (Roed et al., 2012; Schelshorn et al., 2012; Whitaker et al., 2012; **Table 2**). The heterodimerization event between GLP1R and GIPR was found to be dose-dependently induced by GLP1 and inhibited by GIP (Schelshorn et al., 2012). Further application of GLP1 and its antagonists to this system showed heterodimerization to occur independently to GLP1R activation, but was induced by ligand binding, with BRET signals reaching a maximum 10–30 s after addition of GLP1 (Schelshorn et al., 2012). More specifically, the heterodimers were suggested to play a role in allosteric modulation with arrestin recruitment. In basal conditions, GLP1R and GIPR are proposed to exist mainly as monomers or homodimers, GLP1, under low concentrations (<30 nM), acts as a high affinity ligand for functioning of GLP1R but switches to become a low affinity ligand to GIPR with high concentrations (>30 nM). This promotes formation of heterodimers with altered G protein coupling resulting in GIP-like activity that can later be dissociated in the presence of GIP (Schelshorn et al., 2012). Heterodimerization of GLP1R and GIPR was also confirmed by a later study with wild-type GLP1R rescuing mutant GIPR function (Whitaker et al., 2012).

### VIP/PACAP RECEPTORS

To elicit physiological response, vasoactive intestinal peptide (VIP) and pituitary adenylate cyclase activating polypeptide (PACAP) bind to three receptors, namely VIPR1, VIPR2, and PACAP receptor type 1 (ADCYAP1R1). Discoveries of VIPR1 and VIPR2 homodimers were made during the study of SCTR oligomerization in 2006 (Harikumar et al., 2006; **Table 1**). These physical interactions were demonstrated by BRET experiments and found to be modulated by agonist but not antagonist binding (Harikumar et al., 2006). In the same study, BRET results also indicated that VIPR1 and VIPR2 to form hetero-oligomers and VIP binding was found to affect this association negatively (Harikumar et al., 2006; **Table 2**). Formation of these oligomeric complexes was also supported by co-immunoprecipitation studies (Langer et al., 2006). Besides formation of hetero-oligomers between the two subtypes, both VIPRs can oligomerize with SCTR. Interestingly, co-expression of SCTR with either VIPR resulted in strong intracellular FRET signals, suggesting the trapping of hetero-oligomers within organelles of the biosynthetic pathway (Harikumar et al., 2006). Since this retention occurred independent to SCT and VIP stimulation, it has been described as a regulatory step, allowing SCTR to have dominant-negative effects by inhibiting VIP’s action on cells expressing both receptors (Harikumar et al., 2006). Within a physiological context, the SCT and VIPR are often co-expressed; therefore information regarding their physical interactions may provide some insights toward diseases such as pancreatic carcinoma (Estival et al., 1981; Ding et al., 2002a). Of the receptor trio, information regarding ADCYAP1R1 oligomerization is scarce with only one study indicating its homodimerization by time-resolved FRET studies (Maurel et al., 2008; **Table 1**).

### GROWTH HORMONE-RELEASING HORMONE RECEPTORS

Growth hormone-releasing hormone (GHRH) is a hypothalamic peptide responsible for stimulating growth hormone release and disruption of the peptide–receptor pair often results in abnormal growth, such as dwarfism or gigantism. GHRHRs have been suggested to form oligomers, with co-expression of mutant and wild-type receptors having dominant-negative effects (McElvaine and Mayo, 2006). The physical interaction amongst GHRHRs was implied with the reduction of ligand binding ability and cyclic adenosine monophosphate (cAMP) production to 60% in presence of truncated receptors (McElvaine and Mayo, 2006). These observations were further supported by detection of both receptor forms by differentially tagged co-immunoprecipitation studies (McElvaine and Mayo, 2006). The relevance of these findings has been hypothesized in the context of pituitary adenomas for example, with preferential expression of the mutant receptor down-regulating signaling and thus modulating growth hormone release (Matsuno et al., 1999).

### PARATHYROID HORMONE RECEPTORS

Parathyroid hormone receptors (PTHRs) play an important role in calcium homeostasis and bone maintenance, mediating the effects of its natural ligands parathyroid hormone (PTH) and PTH-related protein (PTHrP). In 2008 and 2009, the crystal structures of PTH and PTHrP bound human PTHR1 were determined by engineering the receptor as a readily crystallizing maltose binding protein (Pioszak and Xu, 2008; Pioszak et al., 2009). A “hot dog bun” three-layer α-β-βα fold structure was described, forming the central hydrophobic groove within which the ligands in an amphipathic helical fashion docked (Pioszak and Xu, 2008; Pioszak et al., 2009). More importantly, the crystal structures depicted ligand binding to PTHR1 ECD monomers in a 1:1 stoichiometric ratio (Pioszak and Xu, 2008; Pioszak et al., 2009). Interestingly a year later, the crystal structure of ligand-free PTHR1 showed the ECDs as dimers (Pioszak et al., 2010). These ECD dimers were formed in a similar fashion to ligand-bound PTHR1, with the C-terminal of one ECD forming α-helices to occupy the peptide-binding groove of the other (Pioszak et al., 2010). Besides crystallography, BRET and FRET experiments also supported existence of these ECD-mediated PTHR1 homodimers (Pioszak et al., 2010). To confirm the ligand-independent nature of ECD dimerization shown by the crystal structures, PTH was applied to PTHR1s in BRET studies and as predicted, led to agonist-induced dissociation of the PTHR1 homodimers (Pioszak et al., 2010). Furthermore, co-expression of mutant and wild-type ECD PTHR1s showed that monomeric units of the PTHR1 were sufficient for functionality (Pioszak et al., 2010). With the discovery of monomeric and dimeric units of PTHR1, this may be useful in understanding how the two biologically distinct peptides PTH and PTHrP are able to modulate function through binding to the same receptor (Pioszak et al., 2010).

### CALCITONIN AND CORTICOTROPIN-RELEASING HORMONE RECEPTORS

Targeting mainly osteoblasts, calcitonin is important in calcium homeostasis and has anorexic and analgesic effects in the central nervous system. In a study focused on rabbit calcitonin receptors (CALCRs), homo- and heterodimers of the alternatively spliced CALCR (∆e13) and CALCR type 1a isoform (CALCR1a) were detected by co-immunoprecipitation and FRET analyses (Seck et al., 2003). Co-expression resulted in a dominant-negative effect of the splice variant ∆e13 over CALCR1a, resulting in reduced CALCR1a surface expression, cAMP response, and extracellular signal-regulated kinase (ERK) phosphorylation (Seck et al., 2003). The combined data highlight the importance of CALCR1a homodimers in proper expression and functioning, and also suggest a role of ∆e13 in regulating CALCR1a expression (Seck et al., 2003). In contrast, both static and saturation BRET studies on the human CALCR were found unable to form homodimers (Harikumar et al., 2010). On the basis of SCTR studies where TM4 was identified to be an important interface (Harikumar et al., 2007), the same TM was also considered for CALCR (Harikumar et al., 2010). Comparing human and rabbit TM4 domains, only one single amino acid was found to differ between the two species, namely Arg and His at position 236, respectively (Harikumar et al., 2010). Synthetic TM4 peptides were constructed by exchange of these identified lipid-facing residues to those from respective CALCR species and human SCTR, and their application enhanced human CALCR BRET signals (Harikumar et al., 2010). Similar to the SCTR, lipid-exposed residues in TM4 are also key in facilitating homodimerization events in human CALCR (Harikumar et al., 2010). Though not studied in-depth, the closely related CALCRL has also been demonstrated to exist as homodimers (Heroux and Bouvie, 2005).

Within the class B GPCRs, the corticotropin-releasing hormone receptors (CRHRs) are the least studied. CRHRs are mediators of stress response, modulating the release of adrenocorticotropic hormone from the anterior pituitary. Two subtypes of the receptors exist, but only subtype 1 (CRHR1) has been studied for oligomerization. Data from FRET and co-immunoprecipitation studies show evidence of CRHR1 forming constitutive homodimers independent to ligand binding but their physiological relevance remains unexplored (Kraetke et al., 2005; Young et al., 2007).

## FUNCTIONAL CONSEQUENCES OF OLIGOMERIZATION IN CLASS B GPCRs

Within the large superfamily of cell surface proteins, obligatory oligomerization is unique only to class C GPCRs and recognized as a prerequisite for activation and allosteric modulation (Kniazeff et al., 2011; Chun et al., 2012). However, in the case of class A and B GPCRs where oligomerization has also been established as a real event, the implications on their functions remain unresolved. This is further complicated by the fact that monomeric GPCRs are demonstrated to be independently capable of regulating signal transduction via a series of processes including the activation of G proteins (White et al., 2007; Whorton et al., 2007, 2008; Kuszak et al., 2009), kinase phosphorylation and signal “switch-off” by arrestin binding (Xu et al., 1997; Bayburt et al., 2011). To decipher the underlying functional significance, the effect of GPCR oligomerization on various functional properties needs to be examined with emphasis on class B GPCRs, as examples.

### LIGAND BINDING

Ligand binding, either with an agonist or antagonist, is fundamental in activating subsequent downstream signaling cascades. Natural ligands for the class B GPCRs share common features, comprising of 25 or more amino acids and have diffuse pharmacophoric domains (Ulrich et al., 1998). Critical residues have been identified throughout the length of the peptides (Dong et al., 2011), with the N-terminus recognized as necessary for receptor binding and signaling, whilst the C-terminus contributes mainly to high affinity binding and receptor specificity. Crystallography (Sasaki et al., 1975; Parthier et al., 2007) and nuclear magnetic resonance (NMR; Grace et al., 2007; Venneti and Hewage, 2011) analyses have also been useful, elucidating the class B ligands to preferentially assume an α-helical conformation particularly in their C-terminal regions. Structure–activity studies have further implicated the specific interaction of ligand–receptor regions, with the C-terminal ligand domain first fitting into the receptor ECD peptide-binding cleft, thus facilitating the N-terminal ligand domain to associate with the receptor core helical bundle domain (Dong et al., 2004, 2008; Mann et al., 2007; Parthier et al., 2009). Though the molecular interactions described are on the basis of monomeric GPCRs, similar associations may be expected from oligomeric forms. Similarly, molecular modeling and crystallization studies have predicted the binding pockets in SCTR and PTHR homodimers, respectively, to be localized in the extracellular N-terminal domains (Gao et al., 2009; Pioszak et al., 2010). Moreover, the SCTR homodimer peptide-binding clefts are demonstrated to reside opposite to the dimerization interfaces that may allow structural freedom for each protomer to bind to its respective ligand in a 1:1 stoichiometric ratio, as expected for monomeric GPCRs (Gao et al., 2009). Though limited, the two models show oligomerization to have minimal structural modifications in terms of adequately exposing the binding pockets, allowing ligand–receptor interactions in class B GPCRs.

In majority of studies for this receptor class, the use of peptide or mutant receptors to inhibit dimerization events have been utilized to explore the contribution of such receptor interactions toward functionality. On the basis of these studies, oligomerization has been demonstrated to have differential effects on the ligand binding affinity of class B GPCRs. Using TM4 peptides that are known to disrupt SCTR homodimerization, both monomeric and dimeric SCTR forms were found to have analogous ligand binding properties (Harikumar et al., 2007). Similarly, oligomerization was also found to have null effects on the ligand binding affinity of PTHR1, with the non-dimerizing R179A/V183A double mutant maintaining wild-type PTH binding activity (Pioszak et al., 2010). In another study, CALCR mutants also bound calcitonin saturably with no statistically significant differences in binding affinity (Harikumar et al., 2010). In contrast, disruption of GLP1R homodimerization by TM4 peptides resulted in elimination of G protein-dependent high affinity binding to GLP1(7–36)NH_2_ (Harikumar et al., 2012). Moreover, co-expression of full-length and truncated GHRHRs resulted in a decrease in GHRH ligand binding and has been described to be resultant of the truncated receptor inducing a dominant-negative effect, leading to conformational changes which inhibit proper binding (McElvaine and Mayo, 2006). Nevertheless, these contrasting findings are consistent with recent reports of oligomerization having variable effects on the kinetics and efficiencies amongst receptors (Dorsch et al., 2009; Hern et al., 2010). Therefore, oligomerization may be important in modulating receptor activity via altering ligand binding affinity only in certain class B GPCRs.

### ALLOSTERY

In recent studies, a link between oligomerization and allosteric regulation has been established in class B GPCRs. Allostery concerns association of a ligand or molecule to the receptor other than its orthosteric site, modifying its functional properties in either a positive or negative manner (May et al., 2007). Using a classical radio-ligand binding approach, the dissociation of labeled SCT in the absence or presence of unlabeled SCT was monitored to study the negative binding cooperativity of SCTRs (Gao et al., 2009). The wild-type dimerizing receptors were found to shift from monophasic to biphasic dissociation upon presence of unlabeled SCT peptide, whilst the non-dimerizing mutant receptors maintained monophasic dissociation, regardless of treatment (Gao et al., 2009). On the basis of these results, the homodimeric SCTRs have been speculated to predominantly exist in a G protein-coupled high affinity state and the monomeric forms in a G protein-uncoupled low affinity state (Gao et al., 2009). Though the molecular basis responsible for the allosteric effects are still unknown, steric hindrance by the two large extracellular amino-terminal domain or selective coupling may be underlying factors contributing to the negative cooperativity observed in SCTRs (Miller et al., 2012). Similarly, the homodimerization event amongst the GCGR and GLP1R have also been identified to exhibit negative cooperativity with addition of labeled native peptides to their respective receptors leading to accelerated dissociation of unlabeled ligands (Roed et al., 2012). More recently, the ECD of the GCGR has been demonstrated to act as a negative regulator of receptor activity through interaction with the third extracellular loop (Koth et al., 2012), a region not only recognized to contribute to receptor affinity and efficacy, but may also aid in optimizing the arrangement of the TM bundle for efficient ligand binding (Peeters et al., 2011). As this case, agonist-occupied homodimers may induce negative cooperativity through structural modifications that affect ligand binding. Though the molecular determinants remain to be confirmed, there is a clear relation between dimerization and negative allostery. Aside from the typical ligand-induced allostery, physical coupling of each protomer in a oligomeric complex may also play a role (Haack and McCarty, 2011). Within the class B GPCRs, this has only been demonstrated in heteromers of the GLP1R and GIPR, where GIPR is suggested to act allosterically on the GLP1R to decrease maximal responses in calcium flux and β-arrestin recruitment assays (Schelshorn et al., 2012). In a later study, mutant GIPR function rescue by wild-type GLP1R was observed, with reduction in GLP1 responsiveness (Whitaker et al., 2012). Again GIPR may be exerting an allosteric effect on GLP1R, but the precise molecular basis needs to be explored with further structural and functional studies.

### EFFECTS OF AGONIST OCCUPATION

Besides allostery, agonist occupation of class B GPCRs can also promote the association or dissociation of oligomeric complexes. In contrast to SCT homodimers that are unaffected by ligand binding (Ding et al., 2002a; Harikumar et al., 2006), GLP1 binding was found to encourage the dimerization between GLP1R and GIPR (Schelshorn et al., 2012). A model proposed describes GLP1 to act as a high affinity ligand for GLP1R at low concentrations, but also becomes a low affinity ligand for GIPR at higher concentrations to trigger formation of the GLP1 and GIPR dimer (Schelshorn et al., 2012). Interestingly, the presence of the high affinity GIP reverses the GLP1 induced association, dissolving the heteromer to a monomeric state (Schelshorn et al., 2012). As previously discussed, the GIPR has allosteric effects on the GLP1R when heterodimerized. Taken together with the agonist-induced association and/or dissociation of receptors, this may be a mechanism by which the receptors activate their G proteins and subsequent downstream signaling cascades (Gomes et al., 2001). Agonist-induced dissociation has also been reported for the PTHR1 via structural mimicry of PTH/PTHrP binding (Pioszak et al., 2010), however the functional relevance remains unclear. Other examples of agonist-induced disruption of oligomerization include VIPR1 and VIPR2 homodimers and VIPR1/VIPR2 heterodimers (Harikumar et al., 2006).

### SIGNALING

Another prominent effect of oligomerization in class B GPCRs is on signaling selectivity and efficiency. In particular, cAMP and PLC are two of the predominant pathways in which physiological responses are elicited through in class B GPCRs. Therefore most oligomerization studies have extended their focus beyond information from ligand binding and BRET studies, but also rely on the cAMP and calcium mobilization responses of receptors. In particular, SCTRs are one of the first class B GPCRs demonstrated to have altered functionality due to homodimerization. By TM4 peptide competition studies, the disruption of SCTR dimerization was found to result in impairment of the generation of cAMP (Harikumar et al., 2007). The dimeric form of SCTR was, therefore, suggested be the optimal conformation for G protein activation (Harikumar et al., 2007). Similarly, disruption of the GLP1R homodimers was found to not only affect cAMP formation and ERK phosphorylation, but also lead to a complete loss of intracellular calcium mobilization response (Harikumar et al., 2012). In the case of GLP1R homodimers, signal transduction involves more than one G protein and/or accessory proteins such as β-arrestins. Nonetheless, dimerization is likely the basis in altering G protein coupling efficiency (Harikumar et al., 2012). This was also evident in the heterodimerization of GLP1 and GIP receptors, with the heteromer having more “GIP-like” (Schelshorn et al., 2012) pharmacological characteristics. Based on most of the studied class B GPCRs, oligomerization seems to exhibit a general trend in affecting G protein activation and signaling efficiency. However, oligomerization should not be a prerequisite for signaling, as monomers such as the PTHRs have been demonstrated to be capable of activating G proteins on their own (Pioszak et al., 2010). Therefore, oligomerization is likely important in providing structural framework to facilitate G protein coupling and thus modulate function.

### TRAFFICKING

A number of studies have suggested the importance of oligomerization in cellular trafficking of the receptors and is a process that occurs early in the biosynthetic pathway (Bulenger et al., 2005). Within the class B GPCRs, this aspect of oligomerization has been most thoroughly explored for the SCTRs. Indeed using a combination of fluorescence and morphologic FRET microscopy studies, the SCTR homodimers were localized within organelles of the biosynthetic pathway including the tubular endoplasmic reticulum, Golgi clusters and the plasma membrane (Lisenbee and Miller, 2006). The homodimerization of SCTRs has therefore been suggested to occur as early as newly synthesized receptors and continue throughout their maturation process (Lisenbee and Miller, 2006). Moreover, non-dimerizing SCTR constructs were not expressed nor yielded a significant morphological FRET signal both intracellularly and on the plasma membrane (Harikumar et al., 2007). Taken together, the SCTR data support the idea that oligomerization may be necessary for receptors to by-pass quality checkpoints before being delivered to their site of action (Bulenger et al., 2005). Consistent with the SCTR data, the VIPRs have also been identified in the biosynthetic pathway and plasma membrane as homo- and heterodimeric complexes (Harikumar et al., 2006). Interestingly, heterodimerization involving the SCTR and VIPR impaired trafficking to the plasma membrane with morphological FRET analyses revealing these heteromers to be trapped within the biosynthetic pathway, lacking cell surface expression (Harikumar et al., 2006). In the case of VIPRs, heterodimerization with SCTR predominates over the homodimerization of VIPRs (Harikumar et al., 2006). This demonstrates dominant-negative inhibition of oligomerization on cellular trafficking, which may be useful for modulating function in cells expressing both the SCTR and VIPR (Harikumar et al., 2006). In a later study, a small amount of mutant GIPR completely lacking *N*-glycosylation and cell surface expression was rescued by co-expression with GLP1R but not GIPR, suggesting the possibility of a heteromer which has a dominant-positive effect in facilitating the trafficking of GIPR (Whitaker et al., 2012).

## STRUCTURAL ELEMENTS GOVERNING OLIGOMERIZATION IN CLASS B GPCRs

With the class B GPCRs key in a number of pathophysiological diseases and mounting evidence indicating their oligomerization to have influence in modulating various functional properties, this has fueled research in unraveling the structural determinants. To date, majority of studies have suggested the importance of different domains in GPCR oligomerization. However within the class B GPCRs, a common theme seems to prevail with several lines of evidence indicating the TM domains to be important. More specifically, it is the fourth TM domain, which has been implicated as a key molecular domain in class B receptor homodimerization (**Table 1**). This has been well documented by the disruption of SCTR homodimerization by TM4 peptides, with mutation studies identifying the lipid-facing residues namely Gly^243^ and Ile^247^ as important interfaces (Harikumar et al., 2007). Similarly, TM4 also provides the primary interface for GLP1R homodimerization, as supported by experimental data with TM4 peptide treatment dissociating the complex (Harikumar et al., 2012). Consistent with findings from the SCTR studies, lipid-facing residues within TM4 of the CALCR were also found to be necessary for dimerization (Harikumar et al., 2010). Though TM4 has been indicated to provide basis for dimerization in class B GPCRs, other domains including the ECD in PTHR (Pioszak et al., 2010) and C-terminal tail in CALCR (Harikumar et al., 2010) may also have some role. More recently, a pseudo signal peptide unique to the CRHR2a was identified to inhibit oligomerization (Teichmann et al., 2012). Steric hindrance by the bulky nature of the high mannose glycan protruding from this N-terminal domain has been suggested, thus preserving CRHR2a as monomers (Teichmann et al., 2012). Current prediction programs are unable to differentiate between canonical and pseudo signal peptides, as a result the presence of similar structures may also exist in other class B GPCRs (Rutz et al., 2006; Teichmann et al., 2012). Taken together, dimerization of class B GPCRs may not necessarily be dependent solely on one structural motif but instead is a highly complex interaction amongst several domains to achieve optimal structural basis for the fine-tuning of physiological processes.

## FUTURE PERSPECTIVES

Over the last three decades, there has been a rapid development of techniques for studying the molecular interaction between GPCRs. Though early evidence was largely overlooked and limited to the class A GPCRS, establishment of RET techniques, in particular, have led to reinterpretation and strongly supports the concept of GPCR oligomerization. In recent years, class B GPCRs are also gaining merit in their ability to form homo- and hetero- oligomers, with the SCTRs being the most extensively studied. Though still nascent, the information available supports the role of dimerization in modulating function of class B GPCRs. Naturally, ligand binding to a receptor results in conformational changes in G protein arrangement to elicit downstream signaling pathways. When GPCRs associate, conformational arrangements also occur to modulate functionality. For example, SCTR homodimers have greater signaling responses than its monomeric counterparts. Furthermore, GPCR dimerization may also act as a repair mechanism, *trans*-complementing domains to restore binding sites and thus signaling, as observed for spliced SCTRs (Ding et al., 2002a,b). In terms of trafficking, oligomerization may also play a role with evidence of SCTR homodimers present as early as nascent molecules throughout the biosynthetic pathway (Lisenbee and Miller, 2006). Though seemingly simple when considering homodimeric units, evidence of GPCR heterodimerization, for example of SCTRs with a wide range of class B GPCRs, such as VIPR and GCGR, suggests a more complex role. Attempts have also been made to link these physical characteristics to pathophysiological responses in class B GPCRs, for example, association of SCTR homodimers to gastrinoma (Ding et al., 2002b) and GLP1R/GIPR heterodimers to diabetes (Whitaker et al., 2012). GPCR oligomerization is therefore functionally relevant and further research will be useful in providing more insights in understanding the molecular determinants to *in vivo* responses and for drug design.

## Conflict of Interest Statement

The authors declare that the research was conducted in the absence of any commercial or financial relationships that could be construed as a potential conflict of interest.
